# Human antibodies targeting ENPP1 as candidate therapeutics for cancers

**DOI:** 10.3389/fimmu.2023.1070492

**Published:** 2023-01-25

**Authors:** Xiaojie Chu, Du-San Baek, Wei Li, Taras Shyp, Brian Mooney, Margaret G. Hines, Gregg B. Morin, Poul H. Sorensen, Dimiter S. Dimitrov

**Affiliations:** ^1^ Center for Antibody Therapeutics, Division of Infectious Diseases, Department of Medicine, University of Pittsburgh Medical School, Pittsburgh, PA, United States; ^2^ Department of Pathology and Laboratory Medicine, University of British Columbia, Vancouver, BC, Canada; ^3^ Department of Molecular Oncology, BC Cancer Research Institute, Vancouver, BC, Canada; ^4^ Canada's Michael Smith Genome Sciences Centre, BC Cancer Research Institute, University of British Columbia, Vancouver, BC, Canada; ^5^ Department of Medical Genetics, University of British Columbia, Vancouver, BC, Canada; ^6^ Abound Bio, Pittsburgh, PA, United States

**Keywords:** therapeutic antibodies, human ENPP1, cancer, CAR T-cells, antibody-drug conjugates (ADC), IgG-based bispecific T-cell engager (IbTE)

## Abstract

Ectonucleotide pyrophosphatase/phosphodiesterase 1 (ENPP1) is a type II transmembrane glycoprotein expressed in many tissues. High expression levels of ENPP1 have been observed in many cancer types such as lung cancer, ovarian cancer, and breast cancer. Such overexpression has been associated with poor prognosis in these diseases. Hence, ENPP1 is a potential target for immunotherapy across multiple cancers. Here, we isolated and characterized two high-affinity and specific anti-ENPP1 Fab antibody candidates, 17 and 3G12, from large phage-displayed human Fab libraries. After conversion to IgG1, the binding of both antibodies increased significantly due to avidity effects. Based on these antibodies, we generated antibody-drug conjugates (ADCs), IgG-based bispecific T-cell engagers (IbTEs), and CAR T-cells which all exhibited potent killing of ENPP1-expressing cells. Thus, these various antibody-derived modalities are promising therapeutic candidates for cancers expressing human ENPP1.

## 1 Introduction

Ectonucleotide pyrophosphatase/phosphodiesterase 1 (ENPP1), also named PC-1, is a type II transmembrane glycoprotein expressed in many tissues including bone and cartilage (1, 2). ENPP1 comprises both nucleotide pyrophosphatase and phosphodiesterase enzymatic activity and plays an important role in purinergic signaling, which regulates immunological, cardiovascular, neurological and hematological functions (3, 4). ENPP1 also serves as the main ectoenzyme responsible for the generation of inorganic pyrophosphates (PPi) while hydrolyzing ATP or GTP to AMP or GMP (5, 6). Hence, loss-of-function mutations of ENPP1 have been associated with many inherited mineralization disorders, including infantile arterial calcification or calcification-related disorders (7–9). Moreover, many studies have shown that ENPP1 also plays an important role in cGAS-STING signaling. Activation of the cGAS-STING pathway, well known as a double-stranded DNA (dsDNA) sensor, induces the expression of immune-stimulated genes and type I IFN in response to infections, inflammation, and cancer (10–12). By interacting with exogenous DNA from tumor cells or endogenous DNA leakage from mitochondria, activated cGAS promotes the formation of cGAMP and further activates STING signaling. By hydrolysis of 2’,3’-cyclic GMP-AMP (cGAMP) in the extracellular space, ENPP1 reduces cGAS-STING signaling, further weakening anti-tumor immune response (13–15).

Additionally, many studies indicate that ENPP1 is highly expressed in many cancers, including lung cancer (16), ovarian cancer (17) and breast cancer (18), and that this overexpression is a strong indicator of poor prognosis in different cancers. Moreover, upregulated ENPP1 has been associated with the promotion of cancer cell migration and bone metastasis in breast cancer (19). Upregulation of ENPP1 also correlates with resistance to immunotherapy and reduction of immune cell infiltration to tumor sites (20). Therefore, loss of ENPP1 activity could suppress metastasis, increase immune cell infiltration, and prolong survival (20, 21). Overall, both ENPP1’s physiological importance and its association with aggressive cancer make it an important target for the development of anti-tumor therapeutics.

One increasingly popular option for targeting overexpressed surface proteins like ENPP1 is antibody therapeutics. Antibody-based immunotherapy is a very attractive and promising therapy for cancer due to its high specificity and affinity for specific tumor cell targets. Many approaches to antibody-based immunotherapies have been developed, including antibody-drug conjugates (ADC), bispecific T cell engagers, and chimeric antigen receptor-modified T cells (CAR T-cells) (22, 23).

ADCs, comprised of a targeting antibody conjugated to anti-cancer small molecule drugs, allow for highly specific delivery of toxic payloads to cancer cells. Using ADCs to target tumors can significantly reduce off-target effects associated with these highly toxic molecules, significantly improving their therapeutic tolerability. Additionally, new approaches based on T cell function, such as CAR T-cells and bispecific T cell engagers, show a promising future for antibody therapeutics. The CAR T mechanism relies on redirecting T cell specificity to a specific tumor antigen by engineering effector T cells to express anti-tumor antibodies on their cell surface. There are currently six CAR T-cell therapies that have been approved by FDA for blood cancers and many CAR T-cells therapies have shown favorable efficacy in clinical trials for the treatment of solid malignancies such as glioblastoma (24–26), non-small cell lung cancer (27), and osteosarcoma (28). However, several, sometimes severe, side effects have been associated with CAR T-cell therapy, including cytokine release syndrome (CRS) and neurologic effects. These potential complications have given rise to another T cell based immunotherapy called bispecific T cell engagers, which combine two antibody constructs with one targeting a tumor-specific antigen and the other binding to effector T cell surface markers, such as CD3. This engager recruits endogenous T cells to the tumor site to attack cancer cells. Compared with the CAR T-cell therapy, the application of bispecific T cell engagers has several unique advantages such as off-the-shelf formats, time-effective generation, reduced cost and less CRS side effects (29).

In the current study, we identified two potent anti-ENPP1 monoclonal antibodies by panning our large human Fab antibody phage-displayed libraries against recombinant ENPP1 proteins. These binders were characterized for their stability and affinity to the target antigen. Among these binders, Fab 17 and 3G12 showed high specificity against ENPP1. ADCs, CAR T-cells, and IgG-based bispecific T cell engager (IbTE) created using these two antibody sequences showed potent killing effects on ENPP1-expressing human hepatoma (HepG2) cells. To our knowledge, this is the first report of ENPP1-specific fully human antibodies as candidates for cancer immunotherapy.

## 2 Materials and methods

### 2.1 Expression and purification of ENPP1, IgG1, Fab and scFv

The ENPP1 extracellular domain was synthesized by IDT (Coralville, IA, USA) and cloned into a pIW-Zeocin expression vector previously created in our lab (30) and fused with a His-tag for purification. To convert Fab antibody candidates to IgG1 format, the heavy chain and light chain of the Fab were amplified and re-cloned into the pcDNA-IgG1 vector. For transient expression of ENPP1-His and IgG1, the plasmid was transfected into Expi293 cells by PEI. The expressed ENPP1 and IgG1 proteins were purified by Nickel resin (GE Healthcare, Chicago, IL, USA) or Protein A resin (GenScript, Piscataway, NJ, USA) respectively. The Fab and scFv binders were expressed in E. coli Top10F’ bacterial cultures with 1mM IPTG induction at 30°C for 16 h. Bacterial pellets were lysed by Polymyxin B (Sigma-Aldrich, St. Louis, MO, USA) and the supernatant was purified on Ni-NTA columns. The other human ENPP family member proteins (ENPP2~ENPP7) were purchased from Thermo Fisher Scientific.

### 2.2 Panning from large Fab phage library

To identify antibody candidates against ENPP1, a large Fab phage library was panned against biotinylated ENPP1-His protein. The antigen was biotinylated by using a biotin conjugation kit (Abcam, Cambridge, UK) according to the manufacturer’s instruction. First, 1 milliliter of the phage library was incubated with streptavidin-coated M-280 Dyna beads (Invitrogen, Waltham, MA, USA) at room temperature for 20 minutes to remove non-specific binders. The phage library was then blocked with 5% milk for 1 hour at room temperature and subsequently incubated with 5ug biotinylated ENPP1-His at room temperature for 1 hour. To separate the antigen-bound phages, the solution was incubated with streptavidin beads at room temperature for 1 hour. The beads were then washed by 0.05% PBST, followed by PBS. The phages were then directly introduced to log phase TG1 bacteria for infection and expression. Two subsequent rounds of panning were performed using progressively lower quantities of the biotinylated ENPP1-His antigen, 2.5 µg in the second round and 1.25 µg in the third. After the third round of panning, 192 individual clones were screened for binding to ENPP1 by ELISA.

### 2.3 Size-exclusion chromatography

The purity and structure of the antibodies were analyzed by Superdex 200 Increase 10/300 GL chromatography (GE Healthcare, Chicago, IL, USA) using methods previously established by our laboratory (31). The standard proteins Ferritin, Aldolase, Conalbumin, Ovalbumin, Carbonic anhydrase and Ribonuclease were used for calibration. A 500 μL sample mix containing the above proteins was loaded into the column and separated by the ÄKTA explorer machine (GE Healthcare, Chicago, IL, USA). For the antibody analysis, 100 μL of filtered antibodies 17 and 3G12 (2mg/mL) in 1 × DPBS (Dulbecco’s phosphate-buffered saline, Gibco, Waltham, MA, USA) were analyzed. Antibodies were eluted by DPBS buffer at a flow rate of 0.5 mL/min.

### 2.4 Enzyme-linked immunosorbent assay

The binding and specificity of Fab, scFv and IgG1 to ENPP1 were analyzed by ELISA as previously described (31). Additional analysis by ELISA was performed in a similar manner with IgG1 to confirm non-specificity with other ENPP members. ENPP1-His protein or other ENPP family proteins were coated at 50 ng/well at 4°C overnight separately, then blocked with 5% milk for 2 hours at room temperature. After washing 3 times by 0.05% PBST, 3-fold serially diluted Fab, scFv, IgG1 binders were incubated on the plate for 1 hour at 37°C. The binding of Fab and scFv candidates was detected by anti-FLAG M2-peroxidase (HRP) antibody (A8592, Sig-ma-Aldrich, St. Louis, MO, USA) while IgG1 candidate binding was detected by HRP-conjugated goat anti-human IgG1 Fc (Sigma-Aldrich, St. Louis, MO, USA) at 1:1000 dilution. After the addition of the secondary antibody, plates were incubated for 1 hour at room temperature. The plates were then washed 3 times by 0.05% PBST. Binding activity was detected using 3,3′,5,5′-tetramethylbenzidine (TMB, Sigma-Aldrich, St. Louis, MO, USA) and was stopped by TMB stop buffer (ScyTek Laboratories, Logan, UT, USA). Absorbance was read at 450 nm.

A competition ELISA was performed using the same coating and blocking procedure. The primary antibody solution contained 13 nM fixed concentration biotinylated IgG 17 with 2-fold serial diluted IgG1 3G12 at 667 nM initial concentration. This solution was loaded onto the plate and incubated for 1 hour at 37°C. Bound biotinylated IgG1 17 was then detected by a secondary streptavidin-HRP antibody incubated for 1 hour at 37°C (P9170, Sigma-Aldrich). The plate was washed 3 times by 0.05% PBST after the secondary antibody incubation, then the reaction was developed by 3,3′,5,5′-tetramethylbenzidine (TMB, Sigma-Aldrich, St. Louis, MO, USA) and stopped by TMB stop buffer(ScyTek Laboratories, Logan, UT, USA. Absorbance was recorded at 450 nm. This experiment was performed in duplicate.

### 2.5 BLItz

The affinity and avidity of the anti-hENPP1 antibody were detected by biolayer interferometry BLItz (ForteBio, Menlo Park, CA, USA) as described previously (31). Briefly, DPBS was used to establish a baseline for 30s, and streptavidin biosensors (ForteBio) were coated with 16.7 μg/mL recombinant ENPP1-Biotin for 2 min. Different doses of IgG1 and Fab were used for association and monitored for 2 min to measure the avidity and affinity. Antigen-coated biosensors with PBS were served as a reference control. Antibody dissociation was monitored in DPBS for 4min. For competition BLItz, DPBS was used to establish a baseline for 30s, and streptavidin biosensors were coated with 16.7 μg/mL recombinant ENPP1-Biotin for 2 min. 200nM of IgG1 3G12 were used for association and monitored for 2min, then 200nM of IgG1 17 were used for continuing association and monitored for 2min, dissociation was monitored in DPBS for 4min.

### 2.6 Membrane proteome array assay

Membrane Proteome Array (MPA) screening was conducted at Integral Molecular, Inc. The MPA is a protein library composed of 6,000 distinct human membrane protein clones, each overexpressed in live cells from expression plasmids. Each clone was individually transfected in separate wells of a 384-well plate followed by a 24h incubation (32). Cells expressing each individual MPA protein clone were arrayed in duplicate in a matrix format for high-throughput screening. Before screening on the MPA, the test ligand concentration for screening was determined on cells expressing positive (membrane-tethered Protein A) and negative (mock-transfected) binding controls, followed by detection by flow cytometry using a fluorescently-labeled secondary antibody. Each test ligand was added to the MPA at the predetermined concentration, and binding across the protein library was measured on an Intellicyt iQue using a fluorescently labeled secondary antibody. Each array plate contained both positive (Fc-binding) and negative (empty vector) controls to ensure plate-by-plate reproducibility. Test ligand interactions with any targets identified by MPA screening were confirmed in a second flow cytometry experiment using serial dilutions of the test antibody, and the target identity was re-verified by sequencing.

### 2.7 Cell lines

Expi 293 cells were purchased from Thermo Fisher Scientific (Waltham, MA, USA) and maintained in Expi 293 expression medium supplemented with 0.4% penicillin-streptomycin (P/S). HepG2 cells, PC3 cells, and 293T cells were purchased from ATCC and were maintained in EMEM, F-12K, and DMEM medium supplemented with 10% FBS and 1% P/S respectively. CHLA10 cells were obtained from British Columbia Cancer Research Centre and were maintained in IMDM medium supplemented with 20% FBS, 1% ITS and 1% P/S. 293T-ENPP1 cells, stably expressing ENPP1, were prepared by transfecting 293T cells with PEI and selected in complete DMEM with 100ug/ml Zeocin (Thermo Fisher Scientific).

### 2.8 Western blot assay

Total protein was extracted from 293T and HepG2 cell lines by RIPA buffer (Thermo Fisher) containing 1× protease inhibitors (Thermo Fisher). 20 μg total proteins of each sample were loaded into a 4 to 12% Bis-Tris mini protein Gel (Thermo Fisher). Subsequently, proteins were transferred to a 0.2 μm PVDF membrane (BioRad). After blocking in 5% non-fat milk at room temperature for one hour, membranes were incubated with rabbit anti-human ENPP1 antibody at 1:1000 dilution (Abcam, EPR22262-75) or β-Actin antibody at 1:1000 (Cell signaling technology) for 4°C overnight. After three washes with TBST, the membranes were incubated with goat anti-rabbit HRP-conjugated secondary antibody (Thermo Fisher) at 1:3000 dilution for 1h at room temperature. After 5 additional washes with TBST, the membranes were visualized by using ECL western blot substrate (Thermo Fisher) and imaged by ChemiDoc XRS+ imaging system (Bio-Rad).

### 2.9 T cell isolation

T cells were isolated from healthy donor’s PBMCs (Zen-Bio, Durham, NC, USA) using the human Pan T cell Isolation Kit (Miltenyi Biotec, North Rhine-Westphalia, Germany) and stimulated with CD3/CD28 T cell activator Dyna beads (Gibco) at 1:1 cell- bead ratio in X-VIVO 15 (Lonza, Basel, Switzerland). The cells were supplemented with 2% human serum (Sigma-Aldrich) and 50IU/ml IL-2 (Miltenyi Biotec). 48h after initiating T cell activation, Dyna beads were removed and the medium was exchanged for TEXMACS (Miltenyi Biotec) including 50IU/ml IL-2 for T cell expansion. The activated T cells were used for the cytotoxicity assay of CAR-T and IgG-based bispecific T cell engager (IbTE) antibodies.

### 2.10 CAR T-cells construction

For the construction of CAR T-cells cells, anti-human ENPP1 scFv 17 and scFv 3G12 converted from Fab format were inserted into the pLVX-IRES-CAR plasmid which was constructed in our lab previously (33). The CAR component consisted of a fully human CD8a followed by 4-1BB and CD3z intracellular domain. To collect lentivirus, 293T cells were seeded at 2×10^6^ in a 10 cm plate one day before transfection. The plasmids encoding anti-ENPP1 CAR, pMD.2G envelope plasmid, and psPAX2 package plasmid were co-transfected into 293T cells by PEI. Lentivirus supernatant was collected after 72h and filtered through a 0.22 μm membrane. Activated T cells were transduced with lentivirus supernatant and 8ug/ml polybrene (Sigma-Aldrich) followed by centrifugation for 90min at 800×g. The centrifugation was set with no acceleration and deceleration and without break. T cells were then incubated at 37°C. After 24h, the medium containing virus was removed and replaced by TEXMACS with IL-2 to expand the T cells. Transduction efficiencies were detected by flow cytometry and CAR T-cells cell function was analyzed 3-4 days later.

### 2.11 IbTE production and antibody drug conjugates generation

For the construction of IbTE, humanized OKT3 was inserted at the C-terminal of IgG1 17 and IgG1 3G12 light chain respectively with (G_4_S)_3_ linker (34). The IbTE proteins were expressed by the Expi293 expression system and purified by protein A resin. For the ADC preparation, the IgG1 17 and IgG1 3G12 were diluted in 30% PPG/DPBS buffer respectively. The payload, Osu-Glu-vc-PAB-MMAE (Levena Biopharma, San Diego, CA, USA) cytotoxic drug, was diluted in DMSO/PPG (1:1) at a concentration of 10mM. The IgG1 binders were conjugated with MMAE at a drug antibody molar ratio (DAR) of 10:1. The reaction was incubated at room temperature for 24 hours with gentle stirring. After incubation, the buffer was exchanged to 0.1% PPG/DPBS by gradually decreasing PPG concentration using Amicon centrifugal filters with MW cutoff of 30kDa (Merck Millipore Ltd.).

### 2.12 Flow cytometry

To detect the cells’ surface expression levels of ENPP1 protein, 2 ×10^5^ cells/test were stained with 100nM anti-ENPP1/PC1 antibody (Abcam, Cambridge, UK) for 30min at 4°C and then stained with Alexa Fluor 488-conjugated goat anti-rabbit IgG H&L at 1:1000 dilution (Abcam) for 30min at 4°C. To determine the cell surface binding of the isolated antibodies, cells were incubated with 100nM hIgG1 17, hIgG1 3G12, 1μM scFv 17 or scFv 3G12, 1μM Fab 17 or 3G12, or IbTE for 30min at 4°C, respectively. An irrelevant IgG1, scFv and Fab were set as isotype controls. Cells were then stained with a secondary antibody, PE-conjugated goat anti-human IgG (Sigma-Aldrich, 1:250) for IgG1 and IbTE or 2μl APC-conjugated anti-FLAG (Miltenyi Biotec) for scFv and Fab. Secondary antibodies were incubated with cells for 30min at 4°C. To examine anti- ENPP1-CAR expression on T cells, CD4^+^ T and CD8^+^ T cells were gated by using 5μl PerCP conjugated anti-human CD3 antibody (Biolegend, San Diego, CA, USA) followed by 5μl APC conjugated anti-human CD4 antibody (Biolegend) and 5μl FITC conjugated anti-human CD8 antibody (Biolegend) separately. CAR expression was examined using 1μg biotinylated ENPP1 followed by PE Streptavidin (Biolegend). For the internalization assay, antibodies were labeled with pHrodo™ Deep Red (Thermo Fisher) following the manufacturer’s instructions. The labeled antibodies (2μg) were then incubated with 2 ×10^5^ of 293T, 293T-ENPP1, and HepG2 cells separately for 2h at 37°C in the cell culture medium. After incubation, cells were washed twice with PBS containing 0.1% BSA. For the receptor detection of internalization assay, the cells were then stained with 2μl Rabbit anti-human ENPP1 antibody (Abcam) for 30min at 4°C followed by the secondary Alexa Fluor 488-conjugated goat anti-rabbit IgG H&L (1:1000) for 30min at 4°C. To determine the cell surface binding of IbTE with T cells, 2 ×10^5^ T cells/test were stained with different concentration of IbTE for 30min at 4°C, the IgG1 17 were set as control. Cells were then stained with a secondary antibody, PE-conjugated goat anti-human IgG (1:250), for 30min at 4°C.

### 2.13 Cytotoxicity assays

The cell-killing activity of anti-ENPP1 CAR T-cells and IbTE were measured by LDH-Glo cytotoxicity assay kit (Promega, Madison, WI, USA) following the manufacturer’s instructions. For the cell-killing activity of anti-ENPP1 CAR T-cells, 5 ×10^3^ cells/well 293T, 293T-ENPP1 and HepG2 target cells were seed in 96-well plate for 24h. Control T or CAR T-cells cells serving as effector cells were then incubated with 293T, 293T-ENPP1, and HepG2 target cells separately at the indicated E: T ratio for 24 hours at 37°C. For the cell-killing activity of IbTE, target cells (1 ×10^4^ cells/well) were seeded for 24h, then mixed with serially diluted IbTE antibodies in 50μl of the growth medium. 50μl of activated T cells were added at the E: T ratio 10:1 or 5:1 and cultured for 24h at 37°C in 5% CO_2_ humidified atmosphere. The final volume for both CAR-T and IbTE was 100 μl/well. The cell supernatant was diluted 100-fold and incubated for 50min for LDH assay set up. Controls for the calculation of percentage cytotoxicity were performed according to the manufacturer’s instructions. The calculation of relative % cytotoxicity is as follows: relative % cytotoxicity = 100 × (Experimental LDH release – medium background)/(target and effector maximum LDH release control – medium Background). For the cell-killing activity of ADC, 293T, 293T-ENPP1, and HepG2 cells were seeded at 2000 cells/well in 96-well white plate and incubated in cell growth medium overnight at 37°C in 5% CO_2_. Serially diluted anti-ENPP1 ADC was added and cultured for 4 days at 37°C in 5% CO_2_. Images were captured under a 4X objective microscope (Olympus microscope, Westborough, MA, USA). Cells-only controls for calculation of percent relative cell viability were included. At the end of the experimental period, the Cell Titer-Glo Luminescent cell viability assay kit (Promega) was used to measure the cell viability following the manufacturer’s instruction. The luminescence were recorded by a synergy^HTX^ multimode reader (BioTek). The calculation of % relative cell viability is as follows: % Relative cell viability = 100 × (Experimental luminescence values – medium Background)/(Control luminescence values – medium Background). To test ADCC, the ADCC reporter bioassay complete kit (Promega) was used. 5 ×10^3^ cells/well 293T-ENPP1 target cells were seeded for 24h, 25µl/well series diluted antibody were added together with 25 µl effector cells from the kit at E: T ratio 20:1 and incubated for 6h at 37°C. The experiment was performed following the manufacturer’s instructions.

### 2.14 Cytokine release assay by ELISA

Control T and CAR T-cells cells were plated in the same manner as they were in the cytotoxicity assays. The supernatant was harvested after culturing cells for 24 hours. Cytokine production was detected by an IFNγ and Granzyme B ELISA kit (Thermo fisher Scientific). The experiment was performed according to the manufacturer’s instructions.

### 2.15 Statistical analysis

Statistical analyses, including EC_50_, were performed by GraphPad Prism. Differences were considered statistically significant when *p*< 0.05. Significance was tested using two-way ANOVA, followed by Tukey’s multiple comparisons test. ****, *p*<0.0001, *, *p*< 0.05.

## 3 Results

### 3.1 Selection of high-affinity Fab antibodies against human ENPP1

**Figure 1:**
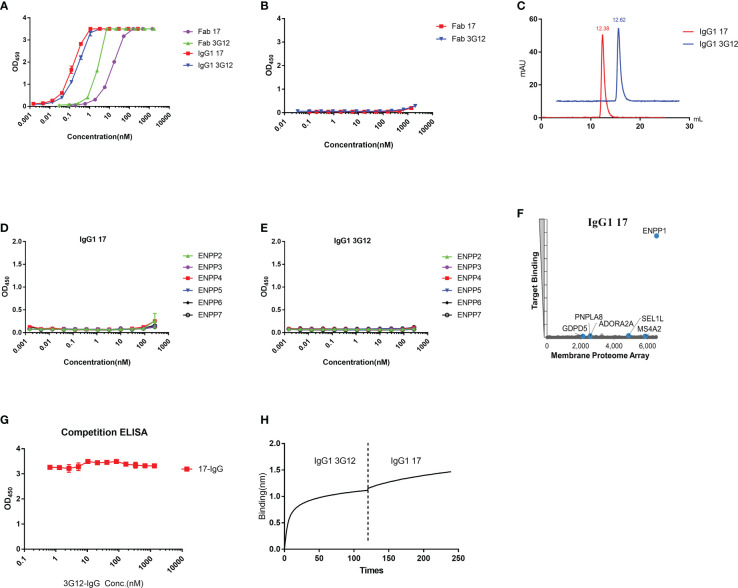
Specificity of Fab/IgG1 with recombinant human ENPP1 and human membrane proteins. **(A)**, Fab 17, Fab 3G12, IgG1 17 and IgG1 3G12 binding to recombinant human ENPP1 measured by ELISA. Experiments were performed in duplicate. **(B)**, Binding of Fab 17 and 3G12 to BSA measured by ELISA. **(C)**, Aggregation evaluation of IgG1 17 and IgG1 3G12 measured by SEC. **(D)**, Binding of IgG1 17 to ENPP other members measured by ELISA. **(E)**, Binding of IgG1 3G12 to ENPP other members measured by ELISA. **(F)**, MPA screen with over 6,000 human membrane proteins. **(G)**, Competitive ELISA of IgG1 17 with serial diluted IgG1 3G12 for binding to ENPP1. **(H)**, Competitive BLItz of IgG1 3G12 with IgG1 17 at 200nM for binding to ENPP1.

In this study, a large phage-displayed human Fab library, developed previously in our laboratory (35) was panned against recombinant human ENPP1 (rhENPP1) protein. After three rounds of panning, several Fab binders were identified. Among these, two antibodies, designated as Fab 17 and 3G12, were selected based on their high affinity, specificity, and desirable biophysical properties. The EC_50_ values of Fab 17 and 3G12, as tested by ELISA, were 13.9 ± 0.36 nM and 2.6 ± 0.06 nM, respectively (**Figure 1A**). Their equilibrium dissociation constant (K_D_) values for binding to rhENPP1, as determined by BLItz, were 61.4 nM and 35.5 nM respectively (**Supplementary Figure S1A, B**; **Table 1**). These two binders did not bind to BSA at high concentrations, demonstrating their binding specificity (**Figure 1B**).

**Table 1:** Blitz results of human ENPP1 antibodies.

Antibody	k_on_ (M^−1^s^−1^)^1^	k_off_ (s^−1^)^1^	K_D_ (nM)^1^
Fab 17	7.2 × 10^4^ ± 1.4 × 10^3^	4.4 × 10^−3^ ± 9.8 × 10^-5^	61.4
Fab 3G12	5.2 × 10^4^ ± 7.5 × 10^2^	1.8 × 10^−3^ ± 4.1 × 10^-5^	35.5
IgG1 17	6.3 × 10^5^ ± 2.5 × 10^4^	4.2 × 10^−3^ ± 2.2 × 10^-4^	6.7
IgG1 3G12	5.2 × 10^5^ ± 2.2 × 10^4^	2.1 × 10^−3^ ± 1.4 × 10^-4^	4.2

^1^Mean kinetic rate constants (k_on,_ k_off_) and equilibrium dissociation constants (K_D_ = k_off_/k_on_) were determined from curve fitting analyses of BLItz results.

To increase antibody avidity and extend their half-lifes, these two Fab binders were converted to an IgG1 format. The EC_50_ of IgG1 17 and 3G12, as tested by ELISA, were 0.14 ± 0.01 nM and 0.24 ± 0.01 nM, respectively (**Figure 1A**). The equilibrium dissociation constants (K_D_) of IgG1 17 and IgG1 3G12, as measured by BLItz, were 6.7 nM and 4.16 nM, respectively (**Figure S1C, D**; **Table 1**). IgG1 17 and 3G12 exhibit monomeric folding without noticeable high molecular weight species (**Figure 1C**) based on size-exclusion chromatography. To further evaluate IgG1 specificity, cross-reactivity with other ENPP family members was assessed by ELISA and a membrane protein array (MPA), a platform for profiling the specificity of antibodies against over 6,000 human membrane proteins. ELISA results showed that IgG1 17 and IgG1 3G12 did not cross-bind to other ENPP family members (**Figures 1D, E**). MPA results showed that IgG1 17, is specific to membrane-associated ENPP1, but also exhibits minor, non-specific binding effects with the human proteins GDPD5, PNPLA8, ADORA2A, SEL1L and MS4A2 (**Figure 1F**). However, after validation, the non-specific effects were found to be caused chiefly by intracellular interaction at a high expression level of these ligands (**Figure S1E** and **S1F**). Competition ELISA and BLItz experiments indicate that IgG1 17 and 3G12 target different epitopes on ENPP1 (**Figures 1G, H**).

### 3.2 hIgG1 17 and 3G12 efficiently bind to ENPP1-expressing cells but exhibit no antibody-dependent cellular cytotoxicity

**Figure 2:**
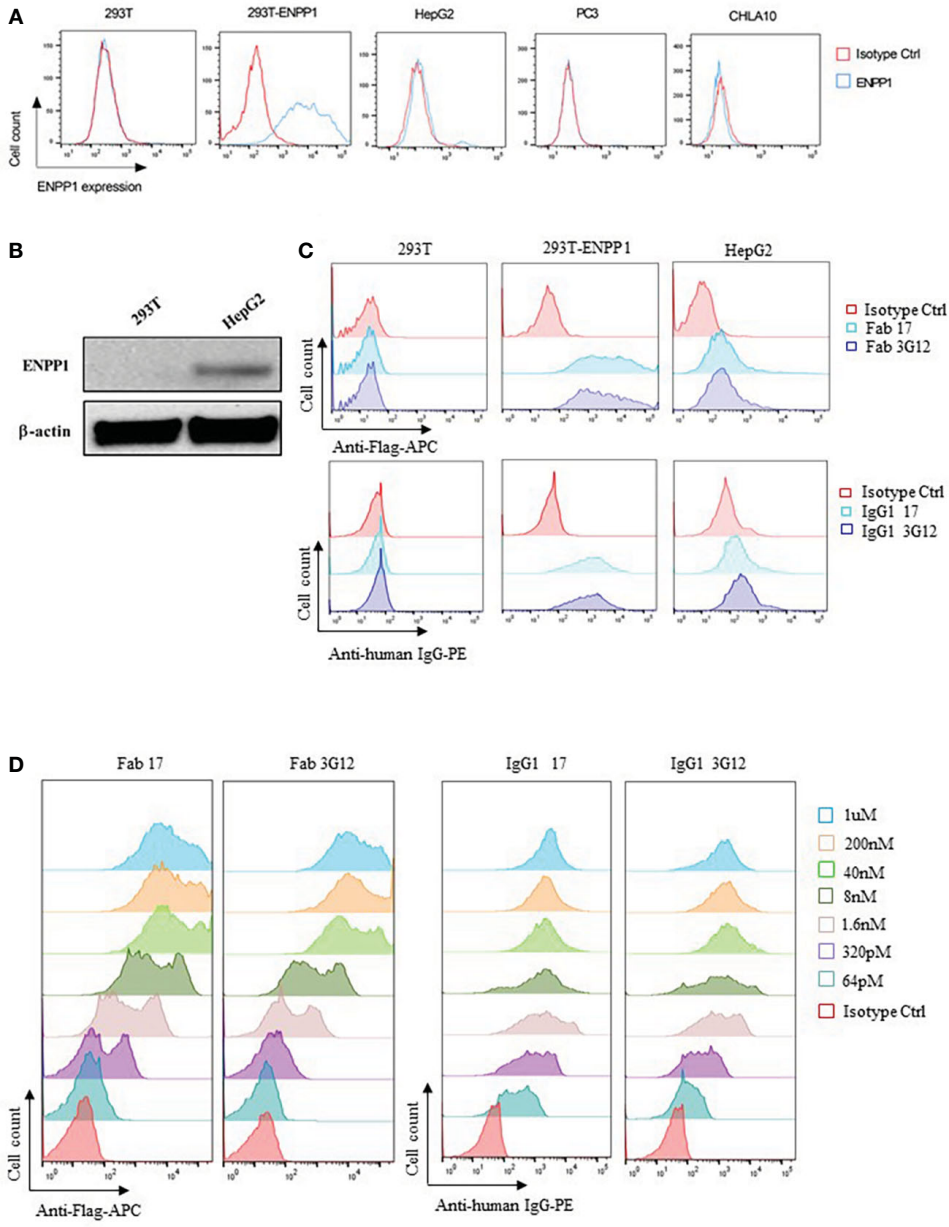
Specificity of Fab/IgG1 with human ENPP1 on cell surface **(A)**, Detection of ENPP1 cell surface expression level on 293T, 293T-ENPP1, HepG2, PC3, and CHLA10 cell lines by a commercial rabbit anti-human ENPP1 antibody followed by an anti-rabbit IgG-AF488 secondary antibody from Abcam. **(B)**, Verification of ENPP1 expression on 293T and HepG2 cells by Western Blot. **(C)**, Cell surface binding of Fab 17 and 3G12 (1uM), IgG1 17 and 3G12 (100nM) on 293T, 293T-ENPP1 and HepG2 cells. **(D)**, Dose-dependent cell surface binding of Fab 17 and 3G12, IgG1 17 and 3G12 on 293T-ENPP1 cells.

To examine the antibody-dependent cellular cytotoxicity (ADCC) of IgG1 17 and 3G12, we first tested the surface expression of ENPP1 on parental HEK293T cells and HEK293T cells isogenically expressing ENPP1 (293T-ENPP1), as well as three different cancer cell lines (human hepatoma cell line HepG2, human prostate cancer cell line PC3, and Ewing Sarcoma cell lines CHLA10) by utilizing a commercial anti-ENPP1 antibody from Abcam (EPR22262-75). Among these cell lines, only 293T-ENPP1 cells showed high expression level (90.1% positive population, the MFI of positive Ab versus isotype is 7534 vs 158). HepG2 cancer cells showed a low but discernable expression level (4.8% positive population, the MFI of positive Ab versus isotype is 146 vs 113), while HEK293T (0.8% positive population, the MFI of positive Ab versus isotype is 289 vs 295), PC3 (1.1% positive population, the MFI of positive Ab versus isotype is 58.6 vs 57.7), and CHLA10 (0.78% positive population, the MFI of positive Ab versus isotype is 38 vs 46.7) showed practically no expression of ENPP1 (**Figure 2A**). The intrinsic ENPP1 expression on HepG2 cells were further verified by Western Blot using the same commercial anti-ENPP1 antibody (**Figure 2B**). Next, we tested if our newly identified anti-ENPP1 binders show affinity for the above cell lines. Results showed that both Fab 17 and 3G12 and IgG1 17 and 3G12 specifically bound to 293T-ENPP1 cells but not to ENPP1 negative 293T cells (**Figure 2C**). Moreover, the Fab and IgG antibodies bound to the 293T-ENPP1 cell in a concentration dependent manner (**Figure 2D**). Most importantly, IgG1 17 and 3G12 showed binding activity with the intrinsically ENPP1 expressing cancer cell line HepG2 (**Figures 2A–C**).

Next, we investigated the ADCC activity of IgG1 17 and 3G12 on ENPP1-expressing cells using an ADCC reporter bioassay, in which the ADCC is detected by the luciferase expression driven by the activation of the NFAT transcription factor in ADCC bioassay effector cells (Jurkat-T CD16A-luc cells) (36, 37). Jurkat-T CD16A-luc cells were co-cultured with target cells 293T-ENPP1 in the presence of serially diluted IgG1 17 and 3G12. Results showed that both IgG1 17 and 3G12 failed to induce significant ADCC, though IgG1 3G12 exhibited a slightly higher signal than that of the control group (**Figure S2**).

Taken together, the specific binding to cell membrane associated ENPP1 by these antibodies warrant their further development as targeting modules in ADC, IbTEs and CAR-Ts for immunotherapy against ENPP1 overexpressing cancers.

### 3.3 Anti-ENPP1 IgG1-MMAE ADC is cytotoxic to ENPP1-expressing cells

**Figure 3:**
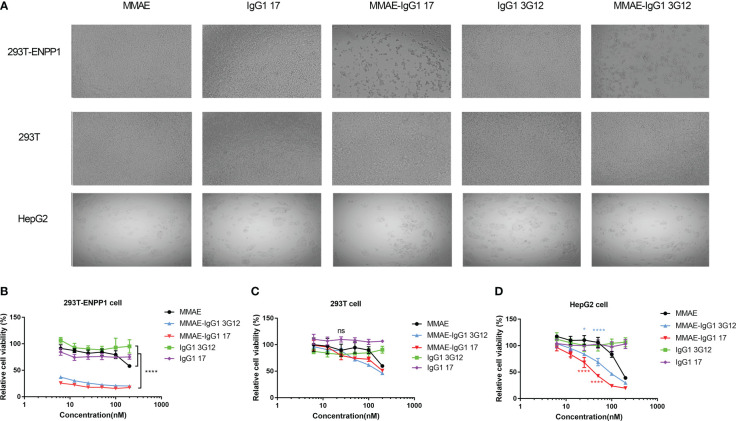
Cytotoxicity of IgG1-MMAE to ENPP1- expressing cells **(A)**, Representative pictures of ADC cytotoxicity, MMAE drug alone or IgG1 alone at 50nM on 293T-ENPP1 cell, 293T cell and HepG2 cell separately. Cells were plated into a 96-well plate and treated with MMAE-IgG1 or IgG1 and MMAE alone at different concentrations for 4 days. B-D, % Relative cell viability of 293T-ENPP1 cells **(B)**, 293T cells **(C)**, and HepG2 cells **(D)** after treatment with MMAE, IgG1 17/3G12 and MMAE-IgG1 17/3G12 separately. Replicates were performed in duplicate. Values are reported as the mean of percent relative cell viability ± SD. Experiments were repeated three times. Significance was tested using two-way ANOVA, followed by Tukey’s multiple comparisons test. ****, *p*<0.0001, *, *p*< 0.05.

To assess the cell cytotoxicity of ADCs based on anti-ENPP1 IgG1, we first investigated IgG1 internalization into 293T-ENPP1 cells by a flow cytometry-based method, in which the fluorescence of a pH-sensitive fluorophore conjugated IgG1 was monitored. The IgG1 17 and 3G12 were efficiently and highly internalized into 293T-ENPP1 cells (**Figure S3A**) and HepG2 cells that intrinsically express ENPP1 (**Figure S3B**). Antibody internalization in HepG2 cells was time-dependent and reached a plateau at 4h incubation (**Figure S3C**). Antibody treatment did not impact the ENPP1 expression level on cell surface (**Figure S3D**). We conjugated MMAE, a potent microtubule inhibitor, to our IgG1 antibody through NHS-lysine coupling. The DAR of MMAE-IgG1 17 was tested by intact protein LC/MS (**Figure S3E**). The MMAE-IgG1 exhibited heterogeneous DARs with the average DAR of 2.28 (**Figure S3F**). We then tested the cell killing by MMAE-IgG1 17 and MMAE-IgG1 3G12 on ENPP1-expressing 293T-ENPP1 and HepG2 cells. MMAE-IgG1 17 and 3G12 showed robust cell killing on 293T-ENPP1 cells in a dose dependent manner (**Figures 3A, B**). The relative cell viability ranged from 17% ~ 26% and 20% ~ 38% for MMAE IgG1 17 and 3G12 at the concentration between 200 nM ~ 6.25 nM (30 μg/ml ~ 0.94 μg/ml) respectively. By contrast, the naked IgG1 and free payload MMAE did not cause substantial cell toxicity. The cell killing effects of IgG1-MMAE seems to be specific and dependent on ENPP1 expression since ADCs did not induce significant cell toxicity on ENPP1 negative 293T cells (**Figures 3B, C**). The fast-dividing 293T cells appear to be sensitive to MMAE, as evidenced by some extent killing effects of free MMAE and MMAE-IgG1 ADC at high concentrations (>100 nM) (**Figure 3C**). We then tested ADC killing effects on HepG2 cancer cells that natively express ENPP1, though HepG2 cells display lower expression of ENPP1 than the 293T-ENPP1 cell line. Both MMAE-IgG1 17 and 3G12 robustly killed HepG2 cells in a concentration dependent manner while the naked IgG1 did not cause cell toxicity (**Figures 3A–D**). Interestingly, it appears that HepG2 cells is more sensitive to the payload MMAE than the 293T cell line.

### 3.4 Anti-ENPP1 IbTE shows potent cytotoxicity against ENPP1-expressing cells

**Figure 4:**
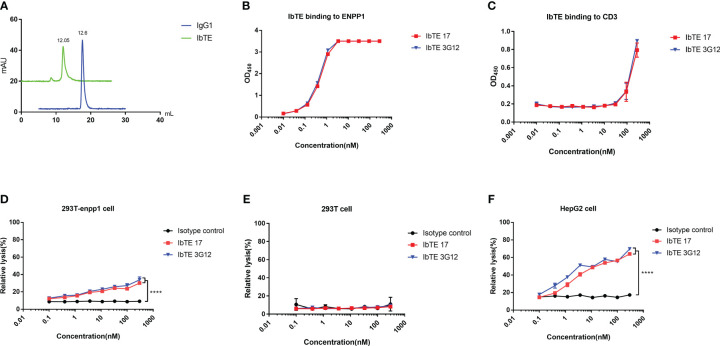
*In vitro* cytotoxic of T cells to ENPP1-expressing cells induced by anti-ENPP1 IbTEs. **(A)**, Aggregation evaluation of IbTE 3G12 and IgG1 3G12 measured by SEC. **(B, C)** IbTE 17 and 3G12 binding to ENPP1 **(B)** and CD3 **(C)** were measured by ELISA. **(D–F)**, Percent relative lysis of 293T-ENPP1 cells by T cells **(D)**, 293T cells **(E)**, and HepG2 cells **(F)** mediated by IbTE 17 and 3G12, respectively. T cells and target cells were added to the 96-well plate at an E:T ratio of 10:1 and simultaneously treated with serially diluted IbTE antibodies for 24h. An irrelevant IbTE isotype control was used. The luminescence signal was detected and used to calculate the percent relative lysis. Experiments were performed in duplicate. Values were reported as the mean of percent relative lysis ± SD. The experiment was repeated three times. Significance was tested by using two-way ANOVA, followed by Tukey’s multiple comparisons test. ****, *p*<0.0001.

To assess the cell cytotoxicity of ENPP1 binder based bispecific T cell engagers, we designed an IgG1 format based bispecific antibody (IbTE), in which the humanized anti-CD3 antibody OKT3 scFv is fused at the C-terminal of IgG1 17 and 3G12 light chain. Proteins were characterized by SEC which confirmed that IbTE was a monomer and has a higher molecular weight (~ 200kDa) than IgG1 (~ 150kDa) (**Figure 4A**). The EC_50_ of IbTE 17 and 3G12 for binding to human ENPP1 as tested by ELISA were 0.5 ± 0.01 nM and 0.4 ± 0.01 nM, respectively (**Figure 4B**). The estimated EC_50_ of IbTE 17 and 3G12 for binding to the recombinant CD3 protein was ~200nM (**Figure 4C**). The IbTE 17 and 3G12 exhibit a dose-dependent binding to T cells as tested by flow cytometry (**Figure S4A**). Next, T-cell mediated cytotoxicity triggered by IbTE was assessed using an LDH assay. Dose-dependent lysis of 293T-ENPP1 cells mediated by IbTE 17 and 3G12 was observed at the E: T ratio of 10:1 and 5:1 (**Figures 4D** and **S4B**), while no toxicity was observed with ENPP1 negative 293T cells (**Figures 4E** and **S4C**). These results indicate ENPP1-targeted killing was, in fact, triggered by these IbTEs. Similar dose-dependent lysis was also found in the HepG2 cancer cell line with estimated IC_50_s for 3.2 ± 0.5 nM and 1.5 ± 0.6 nM for IbTE 17 and 3G12, respectively (**Figure 4F** and **S4D**).

### 3.5 2^nd^ generation anti-ENPP1 CAR T cells mediate cytotoxicity of ENPP1-expressing cells

**Figure 5:**
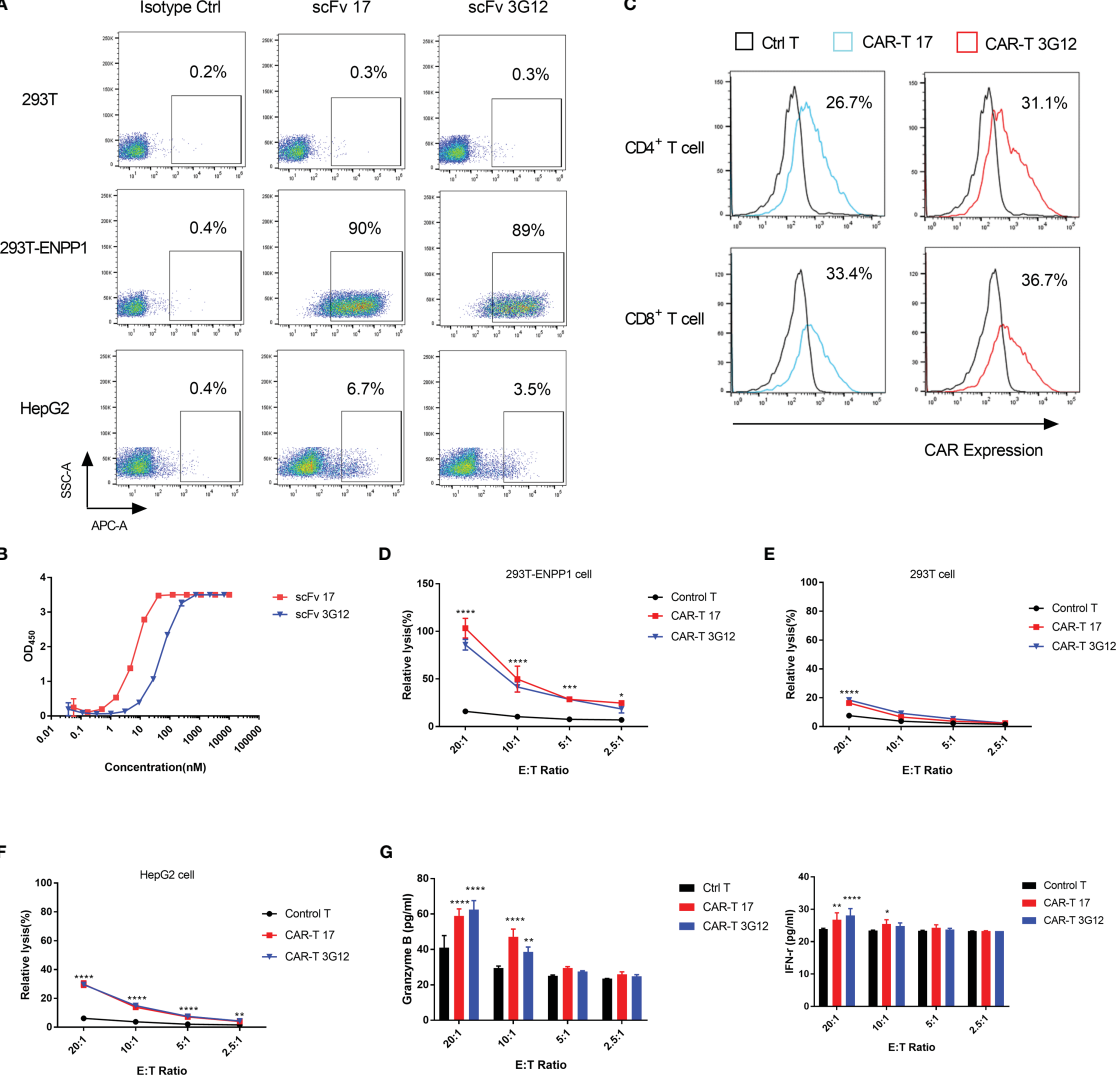
*In vitro* cytotoxicity of CAR T-cells to ENPP1-expressing cells **(A)**, Cell surface binding of scFv format antibodies 17 and 3G12 on 293T, 293T-ENPP1, and HepG2 cells. **(B)**, scFv 17 and 3G12 binding to recombinant human ENPP1 measured by ELISA. Experiments were performed in duplicate. **(C)**, CAR expression levels on CD3^+^CD4^+^ and CD3^+^CD8^+^ T populations of anti-ENPP1 CAR T-cells. **(D–F)**, Percent relative lysis of anti-ENPP1 CAR 17 and 3G12 T cells against 293T, 293T-ENPP1 and HepG2 cells, respectively. **(G)**, Granzyme B and IFN-γ secretion in cell supernatants from control T or CAR T cells after 24h incubation with 293T-ENPP1 target cells at different ratios, as detected by ELISA. Values are reported as means ± SD. The experiment was repeated three times. Significance was tested by using two-way ANOVA, followed by Tukey’s multiple comparisons test. ****p<0.0001. ***p<0.001, **p<0.01, *p<0.05.

To assess the cytotoxicity of our anti-ENPP1 antibodies in the context of CAR T-cell therapy, we generated a lentivirus based 2^nd^ generation CAR vector which encodes a co-stimulatory intracellular domain, a transmembrane domain, and a single-chain variable fragment (scFv) derived from IgG1 17 and 3G12. To confirm that the scFv format maintains binding to ENPP1, we investigated cell binding affinity of scFv 17 and 3G12. Results showed that both scFv retained specific binding to 293T-ENPP1 cells and HepG2 cells, but not to 293T cells (**Figure 5A**). The EC_50_ values of scFv 17 and scFv 3G12 as tested by ELISA were 6.4 ± 0.25 nM and 52.2 ± 1.83 nM, respectively (**Figure 5B**).

Next, we transduced T cells with 2^nd^ generation CARs-encoding lentivirus and confirmed CAR expression by flow cytometry. As shown in **Figure 5C**, the expression level of CAR 17 in transduced CD4^+^ T cells was 26.7% and 33.4% in CD8^+^ T cells, and expression levels of CAR 3G12 in transduced CD4^+^ T cells was 31.1% and 36.7% in CD8^+^ T cells. To determine the cytotoxicity of these anti-ENPP1 CAR T-cells *in vitro*, CAR 17 and CAR 3G12 T cells were co-cultured with ENPP1-positive cells (293T-ENPP1 and HepG2) at E: T ratios of 20:1, 10:1, 5:1 and 2.5:1 for 24 hours. Both CAR T-cell populations showed higher lysis against 293T-ENPP1 and HepG2 cells than untransduced CAR T cells serving as a control group (**Figures 5D–F**). The lower lysis against HepG2 cell than 293T-ENPP1 by CAR T-cells is consistent with the lower ENPP1 expression we observed. The killing appears to be specific, given that the killing of the ENPP1-negative 293T cells (**Figure 5E**) was minimal even at high E:T ratios. This evidence supports the specificity of ENPP1-targeting CAR T-cells. To further analyze the *in vitro* mechanism of CAR T-cell mediated cytotoxicity, Granzyme B and IFN- γ secretion levels were assessed after a 24 hour co-incubation of CAR T-cells with 293T-ENPP1 cells at different E:T ratios. Granzyme B secretion was significantly elevated while IFN-γ was moderately but significantly elevated at an E:T ratio of 20:1 and slightly elevated at 10:1 compared with the control group (**Figure 5G**). Together, these data support the utility of targeting ENPP1 expressing tumor cells with ENPP1-specific CAR T cells.

## 4 Discussion

ENPP1 is a type II transmembrane glycoprotein with pyrophosphatase and phosphodiesterase activity that is expressed in many tissues such as bone and cartilage (2), though expression levels are relatively low in normal human tissue (http://www.proteinatlas.org/ENSG00000197594-ENPP1/tissue). ENPP1 is well known for its function in regulating bone mineralization (38, 39) and modulating insulin (40, 41) and purinergic signaling (42, 43). In addition, recent studies indicate that ENPP1 is also involved in cGAS-STING signaling which plays an important role in the anti-tumor immune response (20). Many studies have shown that ENPP1 is overexpressed in diverse cancer types such as lung cancer (16), breast cancer (19), and ovarian cancer (17). Other cancers including thyroid, liver, head and neck, stomach, carcinoid, pancreatic, testis, and melanoma also display upregulation of ENPP1 RNA or protein levels, as described in the human protein atlas database (http://www.proteinatlas.org/ENSG00000197594-ENPP1/pathology). More importantly, ENPP1 is also associated with poor response to chemotherapy in breast cancer (44). Prior studies have shown that knockdown of ENPP1 can reduce metastasis, induce cell death, and enhance tumor responsiveness to immune checkpoint therapy (20, 45). Overall, the current research shows that ENPP1 could serve as a powerful target for treating a wide range of cancers.

In this study, we generated and characterized two fully human anti-ENPP1 antibodies designated as Fab 17 and Fab 3G12. Both antibodies showed high EC_50_ levels and K_D_ affinities in the nanomolar range. After conversion to IgG1, the avidities of these antibodies were enhanced ~10-fold (**Figure 1** and **S1**). Moreover, these two antibodies were able to specifically bind to human ENPP1, but not BSA and other ENPP family members. MPA results further support the binding specificity of IgG1 17, showing low, non-specific binding with membrane proteins at exclusively high concentration of IgG1 (**Figure S1E, F**). Competition ELISA and BLItz results showed that IgG1 17 and IgG1 3G12 did not compete with each other, indicating that the two antibodies have different binding epitopes on ENPP1 (**Figures 1G, H**). Considering that the two antibodies have the same heavy chain but different light chains, we can infer that the light chain plays an important role in the epitope binding, contributing to the “paratope” for ENPP1 binding. The exact epitope targeting requires further investigation. As ENPP1 also plays an important role in the cGAS-STING pathway by the ENPP1 enzyme activity function, we tested the inhibition effects of our two IgG1 antibodies on ENPP1 enzyme activity (data not shown). We did not observe any inhibitory effects using our antibodies, indicating that the antibody binding epitope is not located in proximity to the ENPP1 enzymatic site. Customized panning using ENPP1 substrates for phage elution could be a possible method to screen antagonistic antibodies in the future.

We tested the killing effects of our antibody-based immunotherapeutics, including ADC, IbTE, and CAR-T, against ENPP1 expressing cells. Both 17 and 3G12-based ADCs showed similar and specific cell-killing effects on ENPP1 overexpressing 293T and intrinsically-expressing HepG2 cells (see **Figure 3**). Additionally, neither ADC exhibited significant killing of ENPP1 negative 293T cells at concentrations lower than 100nM. It is worth mentioning that non-specific killing effects were observed at the highest concentration of 200nM, either in the ADC group or using free MMAE, indicating that the use of high payload concentrations may cause off-target killing. Characterization on tumor cells with different ENPP1 expression levels and comparison with an irrelevant ADC as a negative control is needed to better understand this phenomenon. Using the IbTE format, we observed higher killing rates of HepG2 compared with 293T-ENPP1 cells, noting no killing effects in ENPP1 negative 293T cells (**Figure 4**). As ENPP1 expression is much higher on 293T-ENPP1 than HepG2 cells, we hypothesize that the underlying mechanism targeting tumor cells is different from that of non-tumor (isogenic 293T) cells. It should also be noted that the proliferation rate of 293T cells is much higher than that of HepG2 cells, which may contribute to the apparent lower killing efficacy of IbTEs. The detailed mechanisms of both ADC and IbTE toxicity that warrant further investigation in the future.

CAR T-cells consisting of scFv binders derived from Fab 17and 3G12 also displayed cell killing, though the conversion of these two Fab binders to scFv appeared to reduce the ENPP1 binding affinity of the scFv format compared to the parental Fab format (**Figure 1A** and **5B**). After generating CAR T-cells and testing their efficacy on the 293T-ENPP1 cells, lysis reached ~80% at an E:T ratio of 20:1. At this high E: T ratio, we also observed ~10% non-specific killing of ENPP1 negative 293T cells. No non-specific killing effects were observed at any other E: T ratios (**Figures 5D, E**). Interestingly, unlike IbTEs, the cytotoxic effects of ENPP1-targeting CAR T-cells were much lower against HepG2 cells, with ~20% killing observed at an E:T ratio of 20:1 (**Figure 5F**). This may be due to the lower affinity of the scFv format of anti-ENPP1 antibodies or low expression of ENPP1 on HepG2 cells.

In summary, we generated two fully human antibodies that show a high affinity and specificity towards human ENPP1. These two antibodies showed potent, specific cytotoxic effects on ENPP1 expressing cells in the context of three different therapeutic modalities: ADCs, IbTEs, and CAR T cells (**Figure S5**). These findings warrant additional investigation of the mechanisms underpinning the efficacy and potency of these three immunotherapeutic modalities on different cancer cell lines and models with varying expression levels of ENPP1. Moreover, the *in vivo* toxicity of the three modalities remains unknown due to the limitations of our research. Deeper characterization of *in vivo* toxicity, specificity, and efficacy on tumor inhibition is needed to more accurately understand these factors. Taken together, these anti-human ENPP1 antibodies show significant promise as antibody–based immunotherapy candidates for cancers that express high levels of ENPP1.

## Data availability statement

The original contributions presented in the study are included in the article/**Supplementary Material**. Further inquiries can be directed to the corresponding authors.

## Author contributions

DD, PS and XC conceived and designed the research; XC identified and characterized antibodies, designed and performed functions assays; D-SB made Fab phage libraries; WL conducted the ADC experiments; MH produced the IgG protein for assay; TS prepared the Ewing sarcoma cells; BM, GM performed the proteomics assay for ENPP1 identification. XC wrote the draft of the article. WL, MH, DD and PS revised the manuscript. All authors contributed to the article and approved the submitted version.
