# Supplementary material for: Human antibodies targeting ENPP1 as candidate therapeutics for cancers
Source: Front Immunol. 2023 Jan 25;14:1070492. doi: 10.3389/fimmu.2023.1070492 (PMC9905232; doi:10.3389/fimmu.2023.1070492)
Supplement: Supplementary file 1 [file DataSheet_1.pdf]

Supplementary Material

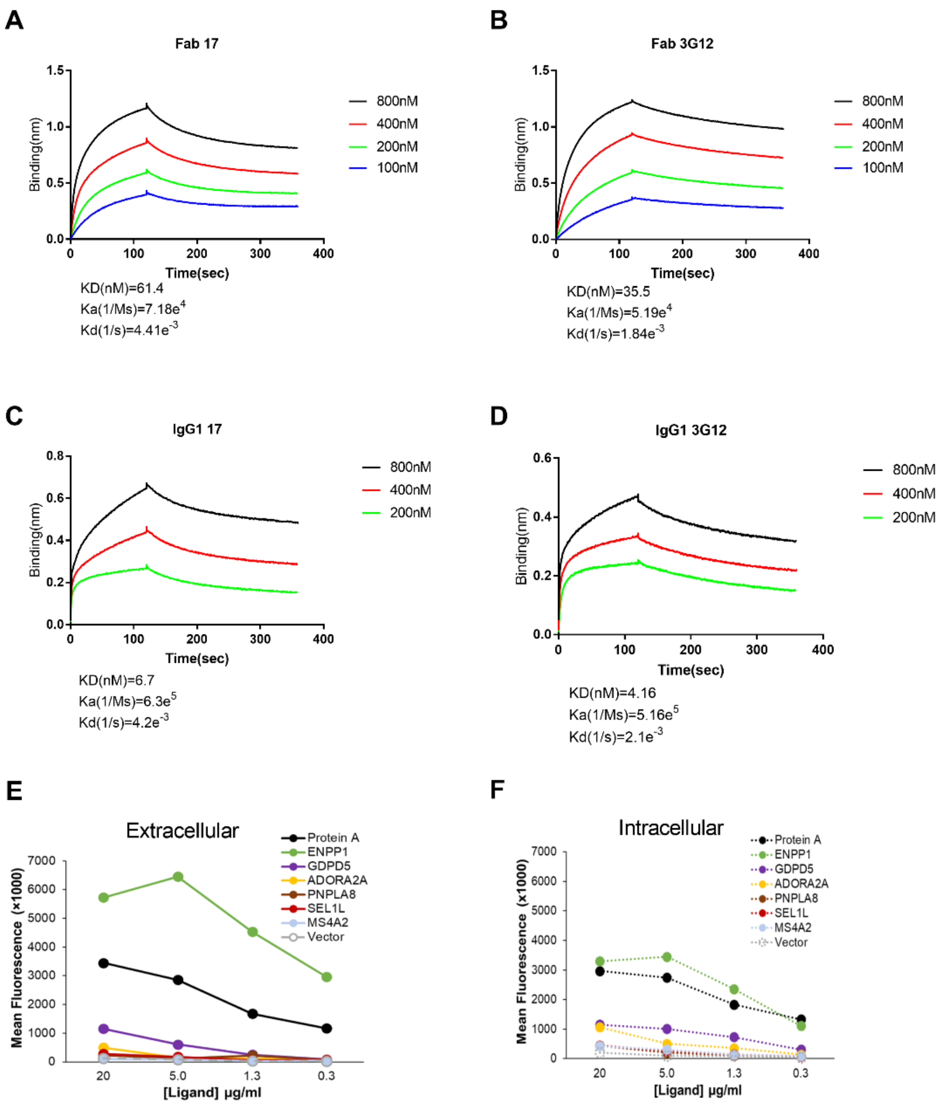

Supplementary Figure 1. Affinity characterization of different format anti-hENPP1 antibodies by Blitz and specificity of IgG1 17 tested by membrane proteome array (MPA).

A-B, Kinetics of Fab 17 (A) and Fab 3G12 (B) binding to recombinant ENPP1 measured by BLItz.

C-D, Kinetics of IgG1 17 (C) and IgG1 3G12 (D) binding to recombinant ENPP1 measured by BLItz.

E-F, Extracellular (E) and Intracellular (F) validation of identified ligand targets by MPA.

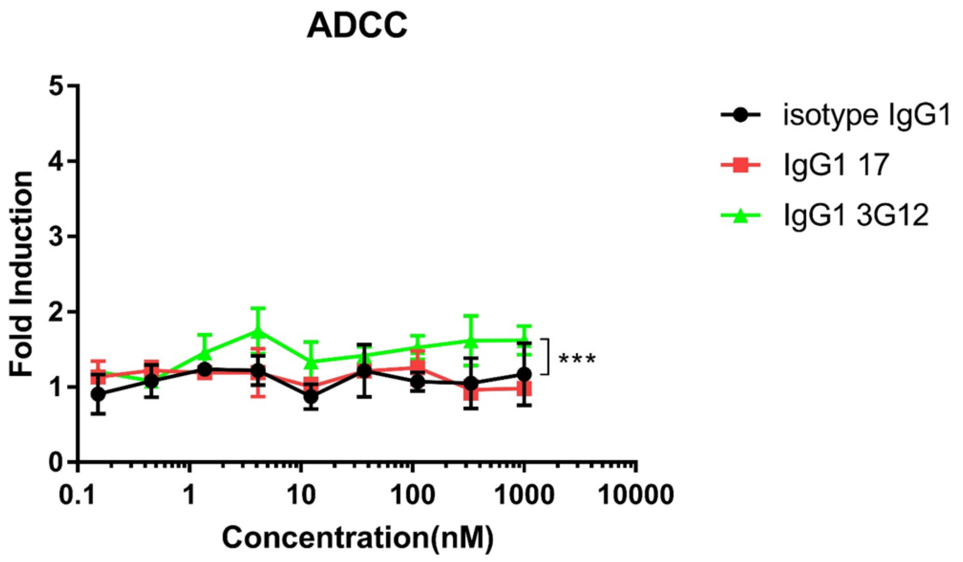

Supplementary Figure 2. ***In vitro*** **ADCC activity characterization**

ADCC activity of anti-ENPP1 IgG1 17, IgG1 3G12, and isotype IgG1 against 293-ENPP1 cell detected by the ADCC reporter bioassay complete kit. Values were reported as the mean ± SD. Significance was tested by using two-way ANOVA, followed by Tukey's multiple comparisons test. ***, $p<0.001$.

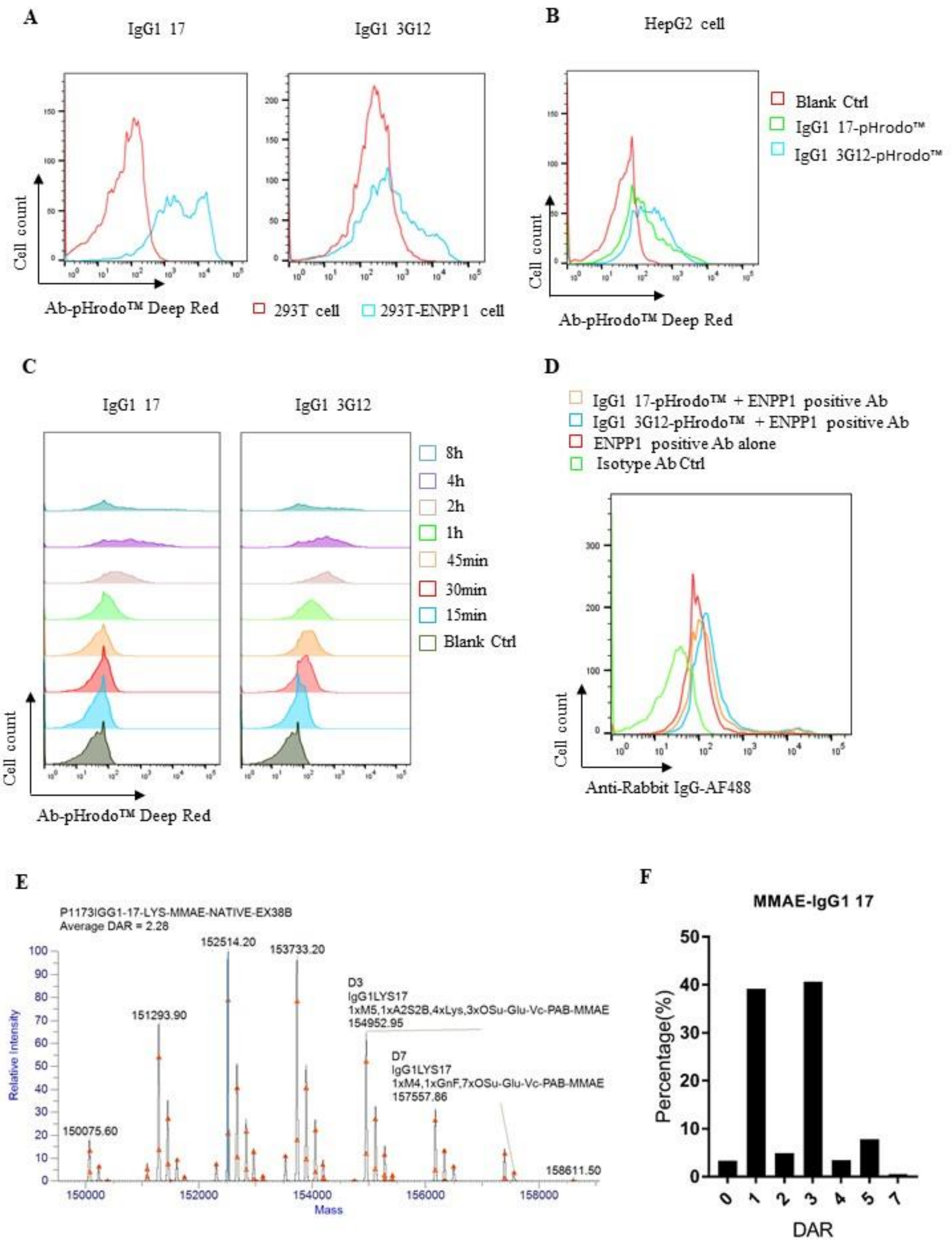
A
IgG1 17
IgG1 3G12
Cell count
Ab-pHrodo™ Deep Red
293T cell
293T-ENPP1 cell
B
HepG2 cell
Blank Ctrl
IgG1 17-pHrodo™
IgG1 3G12-pHrodo™
Cell count
Ab-pHrodo™ Deep Red
C
IgG1 17
IgG1 3G12
8h
4h
2h
1h
45min
30min
15min
Blank Ctrl
Cell count
Ab-pHrodo™ Deep Red
D
IgG1 17-pHrodo™ + ENPP1 positive Ab
IgG1 3G12-pHrodo™ + ENPP1 positive Ab
ENPP1 positive Ab alone
Isotype Ab Ctrl
Cell count
Anti-Rabbit IgG-AF488
E
P1173IGG1-17-LYS-MMAE-NATIVE-EX38B
Average DAR = 2.28
Relative Intensity
150075.60
151293.90
152514.20
153733.20
D3
IgG1LYS17
1xM5,1xA2S2B,4xLys,3xOSu-Glu-Vc-PAB-MMAE
154952.95
D7
IgG1LYS17
1xM4,1xGnF,7xOSu-Glu-Vc-PAB-MMAE
157557.86
158611.50
Mass
F
MMAE-IgG1 17
Percentage(%)
DAR

Supplementary Figure 3. Internalization of IgG1 by ENPP1 positive and negative cell lines

A, The internalization efficiency of IgG1 17 and IgG1 3G12 on 293T cells and 293T-ENPP1 cells detected by Flow cytometry. The IgG1 17 and 3G12 labeled with pHrodo™ Deep Red were incubated with 293T and 293T-ENPP1 cells separately at 37℃ for 2h; B, The internalization efficiency of IgG1 17 and 3G12 on HepG2 cells after incubation at 37℃ for 2h; C, The internalization efficacy of IgG1 17 and 3G12 at different incubation times on HepG2 cells; D, The changes of ENPP1 level on HepG2 cells after antibody internalization tested by the commercial rabbit anti-human ENPP1 antibody; E, Deconvoluted Spectrum from Intact protein analysis of MMAE-IgG1 17. Deconvoluted Spectrum range from 148 kDa to 159 kDa showing the glycan forms with MMAEs detected; F, Percentage of Drug Antibody Ratio (DAR) in the MMAE-IgG1 17 sample tested by intact protein LS/MS, the average DAR=2.28.

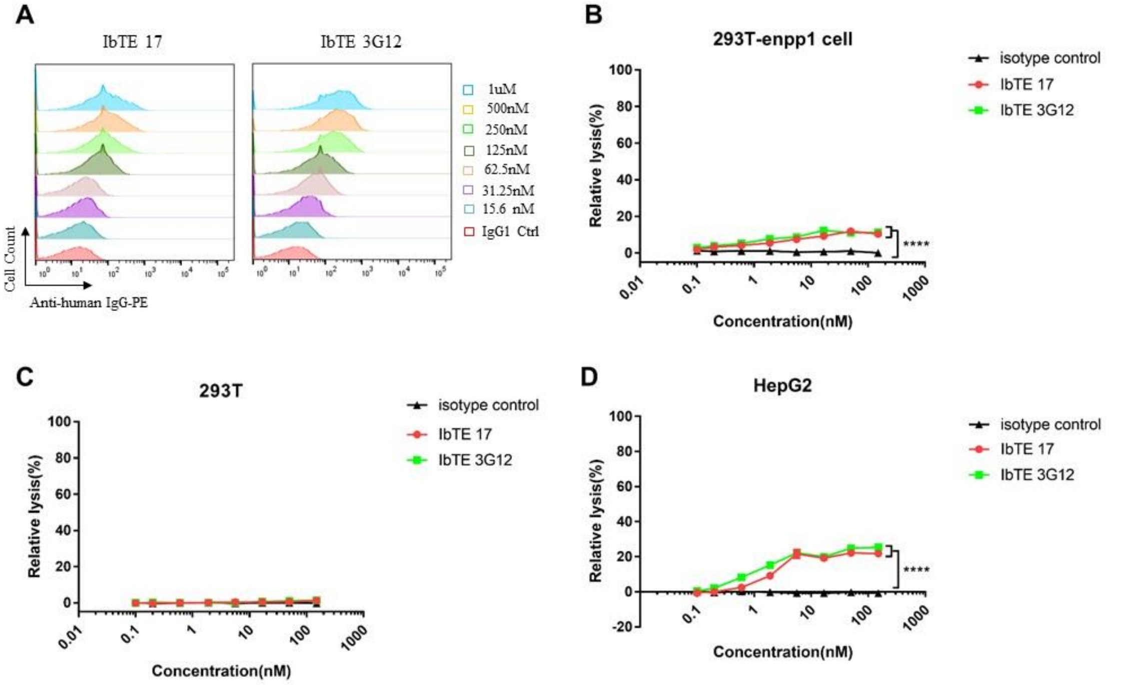

Supplementary Figure 4. ***In vitro*** **cytotoxic of T cells to ENPP1-expressing cells induced by anti-ENPP1 IbTEs.**

A, IbTE binding effects with T cells at dose-dependent manner tested by Flow cytometry. B-D, Percent relative lysis of 293T-ENPP1 cells by T cells (B), 293T cells (C), and HepG2 cells (D) mediated by IbTE 17 and 3G12, respectively. T cells and target cells were added to the 96-well plate at an E:T ratio of 5:1 and simultaneously treated with serially diluted IbTE antibodies for 24h. The luminescence signal was detected and used to calculate the percent relative lysis. Experiments were performed in duplicate. Values were reported as the mean of percent relative lysis ± SD. Significance was tested by using two-way ANOVA, followed by Tukey's multiple comparisons test. ****, $p<0.0001$.

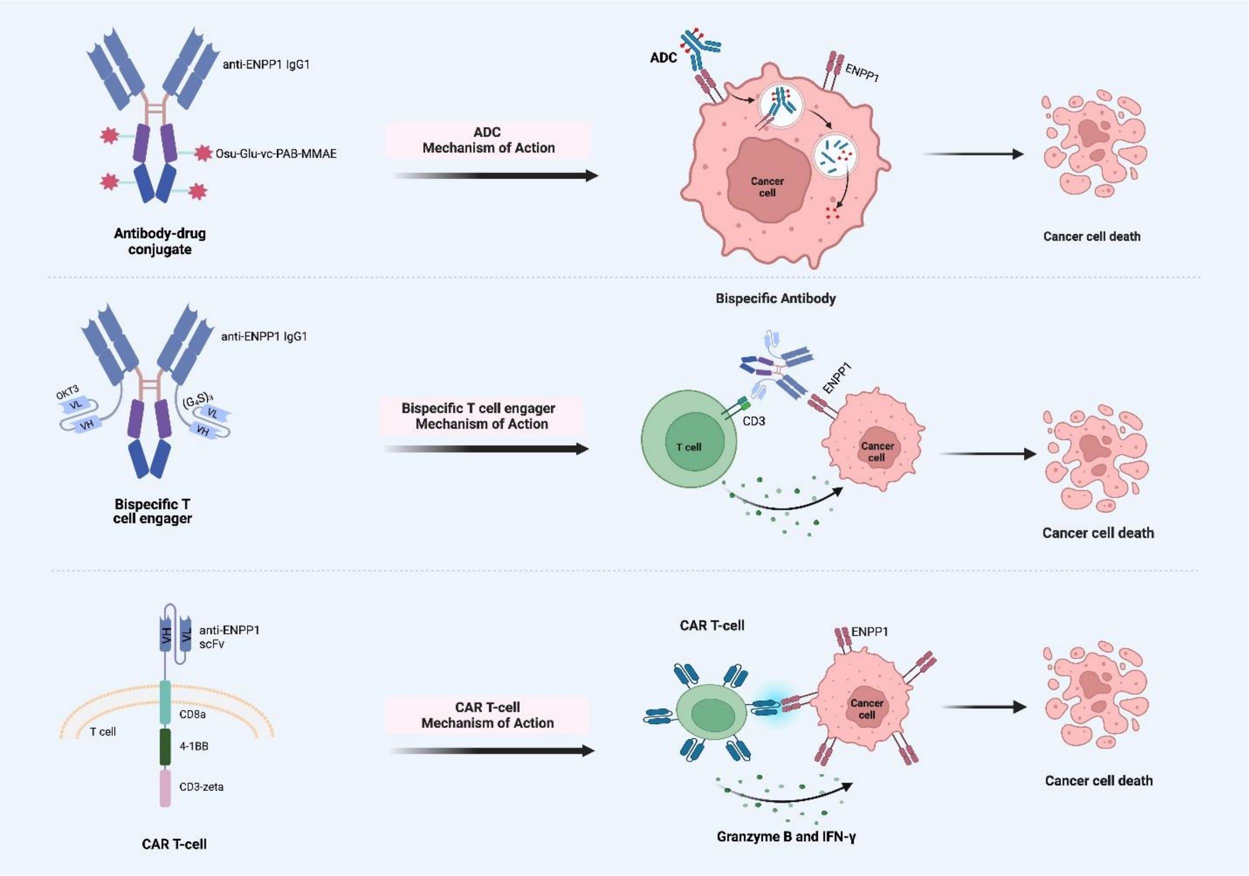

Supplementary Figure 5. Schematic design of the ADC, IgG-based bispecific T cell engager and CAR-T cells. The schematic figure was created with BioRender.com.

Supplementary Method:

Intact protein LC/MS

DAR Analysis of MMAE-lysine-IgG1 17 by intact protein LC/MS method was conducted at Poochon Scientific, Inc. The Intact Mass analysis was performed with a Thermo Scientific Orbitrap Exploris 240 Mass Spectrometer and a Thermo Dionex UltiMate 3000 RSLCnano System at Protein mode. Protein sample was loaded onto a C4 trap cartridge at a flow rate of 20 µL/min. The trapped proteins were eluted onto a reversed-phase Easy-Spray Column MAbPac RP column, 4 µM, 1500A, 150 µm × 150 mm (Thermo Scientific) using a linear gradient of acetonitrile (2-80%) in 0.1% formic acid. The elution duration was 45 min at a flow rate of 1.5 µl/min. Eluted proteins from the Easy-Spray column were ionized and sprayed into the mass spectrometer, using a Nano Easy-Spray Ion Source (Thermo) under the following settings: spray voltage, 1.8 kV, high pressure, Capillary temperature, 275°C. Other settings were empirically determined. Raw data files were analyzed against target protein sequence database using the BioPharma Finder 4.0 software (Thermo, San Jose, CA). Deconvolution Mass Tolerance was set to 20 ppm, whereas Merge Tolerance was set to 30 ppm. Variable Modifications including MMAE on K with an average mass 1334.6 Da and all N-linked glycan forms from the BioPharma Finder 4.0 glycan database.
